# Novel Effect of Berberine on Thermoregulation in Mice Model Induced by Hot and Cold Environmental Stimulation

**DOI:** 10.1371/journal.pone.0054234

**Published:** 2013-01-15

**Authors:** Jing-Fei Jiang, Yu-Gang Wang, Jun Hu, Fan Lei, Michael M. Kheir, Xin-Pei Wang, Yu-Shuang Chai, Zhi-Yi Yuan, Xi Lu, Dong-Ming Xing, Feng Du, Li-Jun Du

**Affiliations:** 1 Protein Science Laboratory of the Ministry of Education, Laboratory of Molecular Pharmacology and Pharmaceutical Sciences, School of Life Sciences and School of Medicine, Tsinghua University, Beijing, China; 2 Perelman School of Medicine, University of Pennsylvania, Philadelphia, Pennsylvania, United States of America; 3 Department of Mathematics, Tulane University, New Orleans, Louisiana, United States of America; University of Wuerzburg, Germany

## Abstract

The purpose of this study was to assess the effects of berberine (BBR) on thermoregulation in mice exposed to hot (40°C) and cold (4°C) environmental conditions. Four groups of mice were assembled with three different dosages of BBR (0.2, 0.4, and 0.8 mg/kg) and normal saline (control). In room temperature, our largest dosage of BBR (0.8 mg/kg) can reduce rectal temperatures (*T*c) of normal mice. In hot conditions, BBR can antagonize the increasing core body temperature and inhibit the expression of HSP70 and TNFα in mice; conversely, in cold conditions, BBR can antagonize the decreasing core body temperature and enhance the expression of TRPM8. This study demonstrates the dual ability of BBR in maintaining thermal balance, which is of great relevance to the regulation of HSP70, TNFα and TRPM8.

## Introduction

Berberine (BBR) is the principal active compound of *Coptidis rhizoma*, which is called *Huang Lian* in Chinese medicine and has been used in clinical practice for thousands of years in China. According to original literature and principles of Chinese medicine, *Coptidis rhizoma* is used in therapies for dispelling heat within the body. It is also used for febrile diseases, such as gastrointestinal infectious disease [1]–[3]. In recent decades, many new pharmacological activities of BBR have been investigated, including anti-inflammatory and anti-apoptotic [4], [5], anti-diabetic [6], [7], anti-hyperlipidemic [8]–[11], and anti-cancer properties [12]–[19], as well as the ability to protect neurons from cerebral ischemia/reperfusion injury by triggering P55γ in the PI3K pathway [20]. Our recent studies have revealed that BBR acts as an intercalator on the TATA box and inhibits gene transcription in a non-specific way [21], indicating that DNA can be a target of BBR in vivo. Throughout our work with BBR, our researchers consistently observed a decreased body temperature. To further understand the potential role of BBR in the regulation of body temperature, the present work comprehensively studied the effect of BBR on environment-dependent thermogenesis in mice. Regulatory thermogenesis by BBR was observed: BBR can antagonize increasing body temperatures in hot environments and, conversely, antagonize decreasing body temperatures in cold environments, which demonstrates a balance in regulation. Moreover, factors such as HSP70 (heat shock protein 70) and TNFα (tumor necrosis factor) for hot stimulation as well as TRMP8 (transient receptor potential cation channel, member 8) for cold stimulation were also observed to be involved in this balance regulation because of their correlation with hot or cold thermal regulation [22], [23].

## Materials and Methods

### Animals

All studies were conducted under protocols approved by the Institutional Animal Care and Use Committee of Tsinghua University and the Animal Welfare and Ethics Committee of Tsinghua University (Approval ID: 2012-DuLJ-BBR). The male ICR mice (8–10 week old,weighing 21–23 g) used in this study were purchased from Vital River Laboratories (Beijing, China) and kept in the animal center of Tsinghua University. Mice were maintained under standard temperature and pressure with 12 h light/dark cycle at a controlled temperature (25°C) and relative humidity (45–55%) with access to standard food pellets and tap water ad libitum.

### Dosages and Groups

Berberine (BBR) was purchased from the National Institutes for Food and Drug Control (Beijing, China) with a purity of 98% (HPLC test). Based on results of previous studies including that of our own laboratory, three dosages of BBR were selected (0.2, 0.4, and 0.8 mg/kg). BBR was administered via intravenous injection. All the thermal detection experiments consisted of all the thermal detection experiments contained 4 groups, which consisted of the three dosage groups of BBR and one control group (normal saline). For electrocardiography (ECG), the dosages used were 0.5, 1, and 10 mg/kg for safety reasons. For motor behavior testing, the mice were given two dosages of BBR, 0.4 and 0.8 mg/kg.

### Experimental Procedures

#### Normal conditions

The mice were kept at room temperature (25°C) and were injected with BBR at three different dosages, 0.2, 0.4, and 0.8 mg/kg. Before BBR injection, the rectal temperature (*T*r) of each mouse was measured using a micro-electronic thermal detector (SN2202 Thermal Detector, Sinan Thermal Instrument, Beijing, China) inserted about 1.5 cm into the rectum and held in position with adhesive tape [24]. Before BBR injection, *T*r was recorded three times in order to determine the average, which was used as the baseline. *T*r was detected and recorded at different points in time, 0.5, 1, 2, 4, 6, 8, and 10 hours after BBR injection. Normal saline was used as a control.

#### Hot conditions

**Figure 1:**
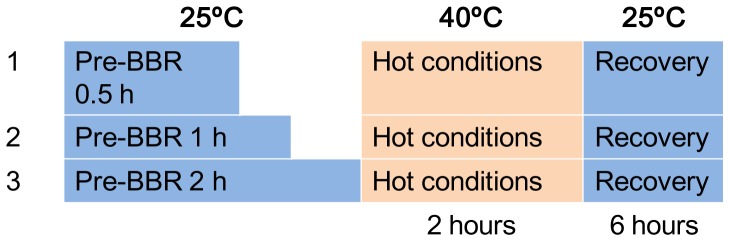
Hot experimental conditions.

The mice were kept at room temperature (25°C) and were injected with BBR at three different dosages (0.2, 0.4, and 0.8 mg/kg). Then, 0.5 to 2 hours after BBR injection, *T*a was detected. In turn, the mice were exposed to different temperatures in a constant thermal incubator (40°C) (TS-1 Incubator, Huangshi Medical Instrument, Hubei, China) for heat conditioning [25]. During this time (2 hours), *T*r was detected every 0.5 hours. After 2 hours of heat conditioning, mice were moved back to a room-temperature environment (25°C). *T*r was assessed continuously for 6 hours. To determine whether BBR exerted any sustained action, the pre-BBR duration was set as 0.5, 1, and 2 hours before heat conditioning. Normal saline was used as a control. The experimental process is shown in Fig. 1.

#### Cold conditions

**Figure 2:**
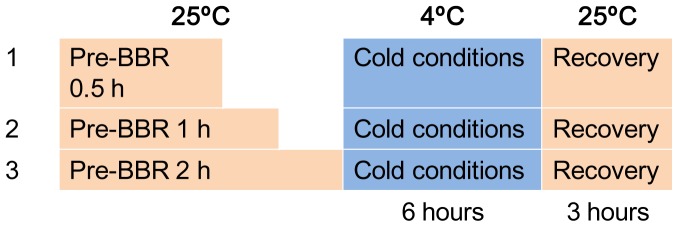
Cold experimental conditions.

The mice were kept at room temperature (25°C) and were injected with BBR at three different dosages (0.2, 0.4, and 0.8 mg/kg). After BBR injection at room temperature at 0.5, 1, and 2 hours, the mice were exposed to cold conditions (4°C) (YC-1 Cold-Incubator, Boyikang Laboratory Instrument Ltd. Beijing, China). Cold conditioning lasted 6 hours [26], [27]. During the cold conditioning, *T*r was detected every hour. After 6 hours of cold conditioning, mice were moved back to a room temperature environment (25°C). *T*r was detected every hour at room temperature after the end of cold conditioning. Recovery time lasted 3 hours. To determine whether BBR exerted any sustained action, the pre-BBR duration was set as 0.5, 1, and 2 hours before cold conditioning. Normal saline was used as a control. The experimental process is shown in Fig. 2.

#### Use of HSP70 inhibitor in hot conditions

ICR mice were divided into five groups: BBR, KNK439, and KNK439 plus BBR were three of the groups. Normal saline was used as a control group (hot stimulation) and then we had one group of normal control mice (normal mice kept at room temperature). Based on prior literature, KNK437 (50 mg/kg) (Santa Cruz, USA) was administered via pre-intraperitoneal injection (i.p.) for 6 hours so that it reached a high concentration in blood [28], [29]. Thereafter, BBR (0.8 mg/kg) was administered via intravenous injection. One hour after BBR injection, mice were kept in hot conditions (40°C) for 2 hours. *T*r was detected every half hour during hot conditions. At the end of hot conditions, mice were killed and their brains were removed. The hypothalamus was isolated and stored at −80°C for mRNA and protein expressions of HSP70.

#### Electrocardiography and motor behavior

Mice were divided into four groups. BBR was injected at three different dosages (0.5, 1, and 10 mg/kg). Our previous work determined that the LD_50_ of BBR was 9.0386 mg/kg [30]. Therefore, the dose of 10 mg/kg was used as the maximum dosage because of its toxicity to the heart by ECG (LCS-2012NK Cardifax, Nihon Koden, Japan) and its effects on cerebral blood flow (CBF) by microcirculation probe (MDL-1 Laser Doppler Analyzer, Nai Kai University, China). Mice were anesthetized by 10% urethane (1 g/kg, i.p.) and kept on the plate in the prone position for 5 min before the experiment. ECG was recorded by the standard II lead. Mouse CBF was detected by putting a laser probe on the bregma [31]. ECG and CBF were recorded prior to BBR injection as control for a within-subjects design. After BBR injection, ECG and CBF were recorded simultaneously at seven different points in time (1, 5, 10, 20, 30, 45, and 60 min). Normal saline was employed as a control.

The motor behavior was assessed via the motor movement and the exploit behavior (SH900 Motor Behavior Instrument, Institute of Meteria Medica, Beijing, China). The mice were allowed to familiarize themselves with a round cage for 2 min before recording. The motor movement was recorded in terms of the number of times each mouse crossed the electric beam-lines in 5 min. The exploit behavior was recorded as the number of times each mouse reared up onto its hind legs in 5 min. Two different dosages were used, 0.4 and 0.8 mg/kg. Then, 0.5 hours after BBR injection, mice were kept in a round cage. Mouse motor movement and exploit behavior were recorded during the first 5 min [32]–[35]. Normal saline was used as a control.

### Real Time PCR and Western Blot

Mice were randomly separated into two groups (the control group with normal saline and BBR group with the dosage of 0.8 mg/kg). As mentioned previously in the experimental procedures of the hot and cold conditions, the brain samples were taken out at different time points. The nuclei of the hypothalamus [36], [37] were isolated quickly from the brains and stored at −80°C until use. In the hot experimental condition, the time points were set at 0, 0.5, 1, 1.5 and 2 hours. In the cold experimental condition, the time points were set at 0, 1, 2, 3, 4, 5 and 6 hours. Mice were injected with BBR 1 hour before hot or cold conditioning. Each time point consisted of six independent mice.

The mRNA levels were determined using SYBR Greenbased quantitative PCR. RNA was prepared by using a RNA simple kit (Tiangen, China) according to the protocol instructions. Total RNA was incubated with DNase to eliminate any residual DNA that might amplify during PCR. From each sample, 30 pg total RNA was reverse transcribed and amplified by using M-MuLV First Strand cDNA Synthesis Kit (Bio Basic, Canada). The Real Mater Mix (SYBR Green) kit (Tiangen, China) was used for PCR. Light Cycler 480II (Roche, USA) was used for detection of the products of PCR. The primer sequence of TRPM8 was designed by Primer Premier 5.0 software and blasted at NCBI GenBank. The primer sequences of HSP70, TNFα and β-actin were used from references, respectively. All primer sequences used in the analyses are produced by Sangon Biotechnology Ltd. (Shanghai, China). The cycling conditions were: 94 °C for 3 min; 45 cycles of 94 °C for 10 sec, 56 °C for 10 sec (TNF-α 58°C, HSP70 57 °C), 72 °C for 10 sec; 72 °C for 10 min and cooled to 4 °C. Data were processed using the Light Cycler 480 SW1.5 software program. β-actin served as the internal control. The primer sequences are as follows: HSP70: sense: 5’- AGCGAGGCTGACAAGAAGAAGGT-3’, antisense: 5’- ACCCTGGTACAGCCCACTGATGAT-3’ [22]; TNFα: sense: 5’- CACCACGCTCTTCTGTCTACTGAACT-3’, antisense: 5’- GGGCTACAGGCTTGTCACTCGAATTT-3’ [22]; TRPM8: sense: 5’- GGTCTTCTCCTGGAACGTGG -3’, antisense: 5’- GTCCCAGGGTGTCCATAACG -3’ generating a 209-bp DNA fragment (GenBank IC number: NM_134252.3); β-actin: sense: 5’-CCCCATTGAACATGGCATTG-3’, antisense: 5’-ACGACCAGAGGCATACAGG-3’ [20].

Total protein was prepared from mouse brain homogenate with 2% SDS. Protein concentration was measured using a Protein Assay Kit (Zhongsheng Biotech., China). Protein (10 µl) was loaded onto SDS-PAGE gels (TNF-α 12%, TRPM-8 and HSP70 10%), and transferred onto nitrocellulose membranes after electrophoresis. The membrane was blocked with 5% bovine serum albumin in PBST (PBS buffer containing 0.1% Tween-20) for 2 h, incubated with primary antibody in PBST overnight at 4 °C. The labeled membrane was washed three times (15 min each) with PBST and then incubated with horseradish peroxidase (HRP)-conjugated secondary antibody in PBST for 1 h at room temperature. The membrane was again washed three times (15 min each) with PBST. The targeted proteins were visualized with the super signal ECL Western blot Substrate (Pierce, China) and the intensity of visualized bands was measured using Quantity One software (Bio-Rad). β-actin was used as an internal control. Data were expressed by the ratio to β-actin. Horseradish peroxidase conjugated secondary antibodies of goat anti-mouse IgG-HRP and goat anti-rabbit IgG-HRP were purchased from Santa Cruz (USA). The primary antibody (monoclonal antibody) of HSP70, TNFα, TRPM8 were purchased from Zhongshan Jinqiao (Beijing, China), Abcam (UK) and Epitomics (USA), respectively. The primary antibody of β-actin was purchased from Santa Cruz (USA).

### Data Analysis

All data are expressed as mean ± S.D. Data were statistically analyzed using one-way analysis of variance (ANOVA) with F value determination. The F test was carried out using Excel software for Office 2007 (Microsoft, USA). The student’s *t*-t test between two groups was performed after the F test. *P* values below 0.05 were considered statistically significant. The data regarding the change in rectal temperature (*T*r) were expressed as Δ*T*r, the difference between *T*r before BBR and *T*r after BBR (Δ*T* = *T*r before BBR- *T*r after BBR). The thermal response index (TRI) (°C. h) was obtained via Graph Pad 5.0 software [38], [39]. The area under the Δ*T* curve (AUC) was defined as TRI [24]. The ordinate and abscissa refer to Δ*T* (°C) and time (hour) of thermal duration, respectively.

## Results

### Thermoregulation of Normal Mice

**Figure 3:**
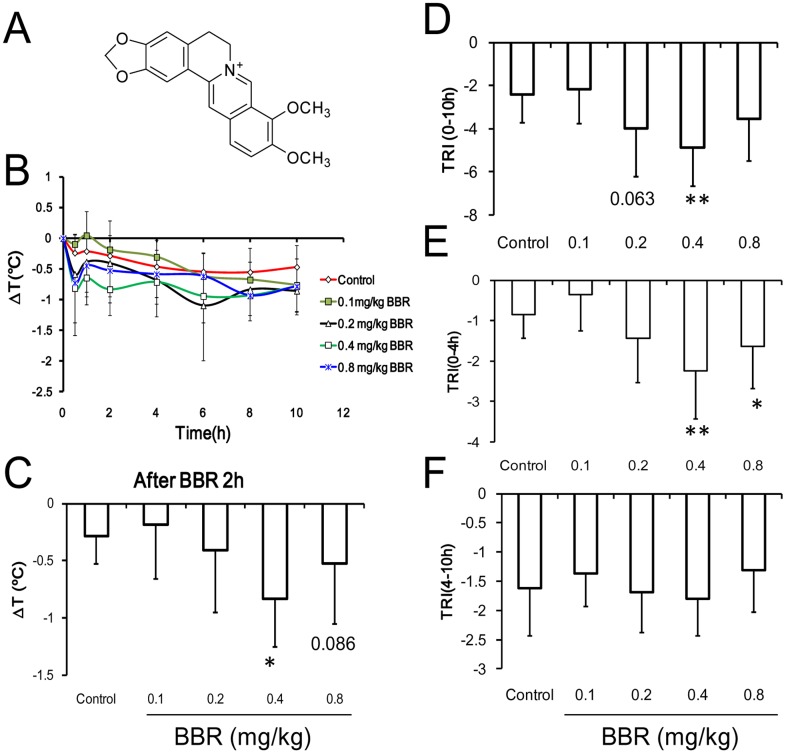
Alteration of core temperature after intravenous administration of berberine (BBR) in normal mice. A: Chemical structure of BBR. B: Time course of temperature changes. C: The temperature at 2-hour time point after berberine injection. F (4, 45) = 3.046, *P* = 0.026. D: Thermal response index (TRI) within 10 hours after berberine injection. F (4, 45) = 3.808, *P* = 0.009. E: TRI within 4 hours after berberine injection. F (4, 45) = 5.404, *P* = 0.001. F: TRI from 6 to 10 hour after berberine injection. F (4, 45) = 0.892, *P* = 0.477. * *v.s.* the control group, *P*<0.05, ** *v.s.* the control group, *P*<0.01. Data were presented as mean ± S.D. from ten independent mice (n = 10).

At room temperature, the body temperatures of mice could be regulated by BBR (Fig. 3A) during the 12 hours after drug administration (Fig. 3B). Within the two hours after drug administration, the body temperature of mice treated with BBR at the dosage of 0.4 mg/kg and 0.8 mg/kg decreased faster than other periods (Fig. 3C). Ten hours after BBR injection, the TRI attenuated in the group given 0.4 mg/kg BBR, but no positive effect was observed in the group given 0.8 mg/kg BBR (Fig. 3D). However, four hours after BBR administration, a decreased TRI was observed both in 0.4 mg/kg and 0.8 mg/kg BBR group (Fig. 3E). TRI stayed at a low level 4 hours after BBR injection, which lasted until 10 hours after BBR injection (Fig 3F). This suggested that BBR can distinctly decrease body temperature 4 hours after injection. BBR was able to decrease the body temperature of normal mice via dosages of 0.4 mg/kg and 0.8 mg/kg (Fig. 3).

### Thermoregulation under Hot Conditions

**Figure 4:**
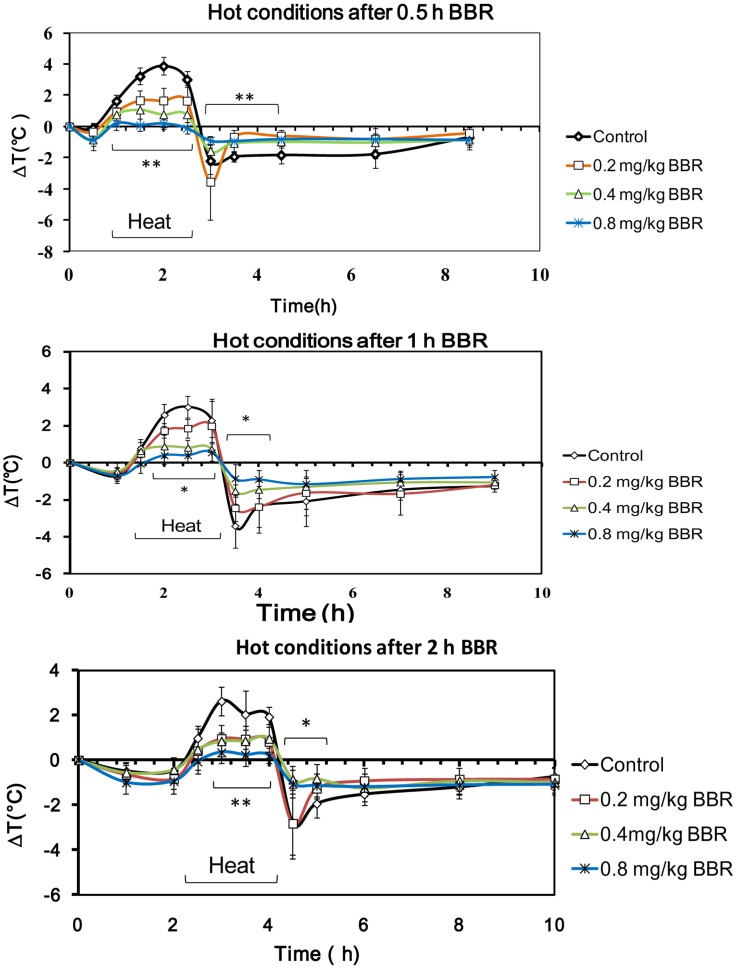
Time course of mouse core temperature after berberine (BBR) injection in hot conditions. Data were presented as mean ± S.D. from ten independent mice (n = 10). * *v.s.* the control group, *P*<0.05. ** *v.s.* the control group, *P*<0.01.

In the hot experimental condition, the body temperature of mice in the control group increased by 3°C on average in comparison to normal conditions (Fig. 4). The increased body temperature was dose-dependently antagonized by BBR. The body temperature of the mice decreased sharply by about 3°C below body temperature of mice under normal condition, and it then increased slowly. BBR at the dosage of 0.4 mg/kg and 0.8 mg/kg was able to antagonize the increasing body temperature and kept the temperature close to normal baseline. Differences in the period before BBR administration (0.5, 1, and 2 hours before conditioning) had no discernible impact on the effect of BBR in its regulation of body temperature.

**Figure 5:**
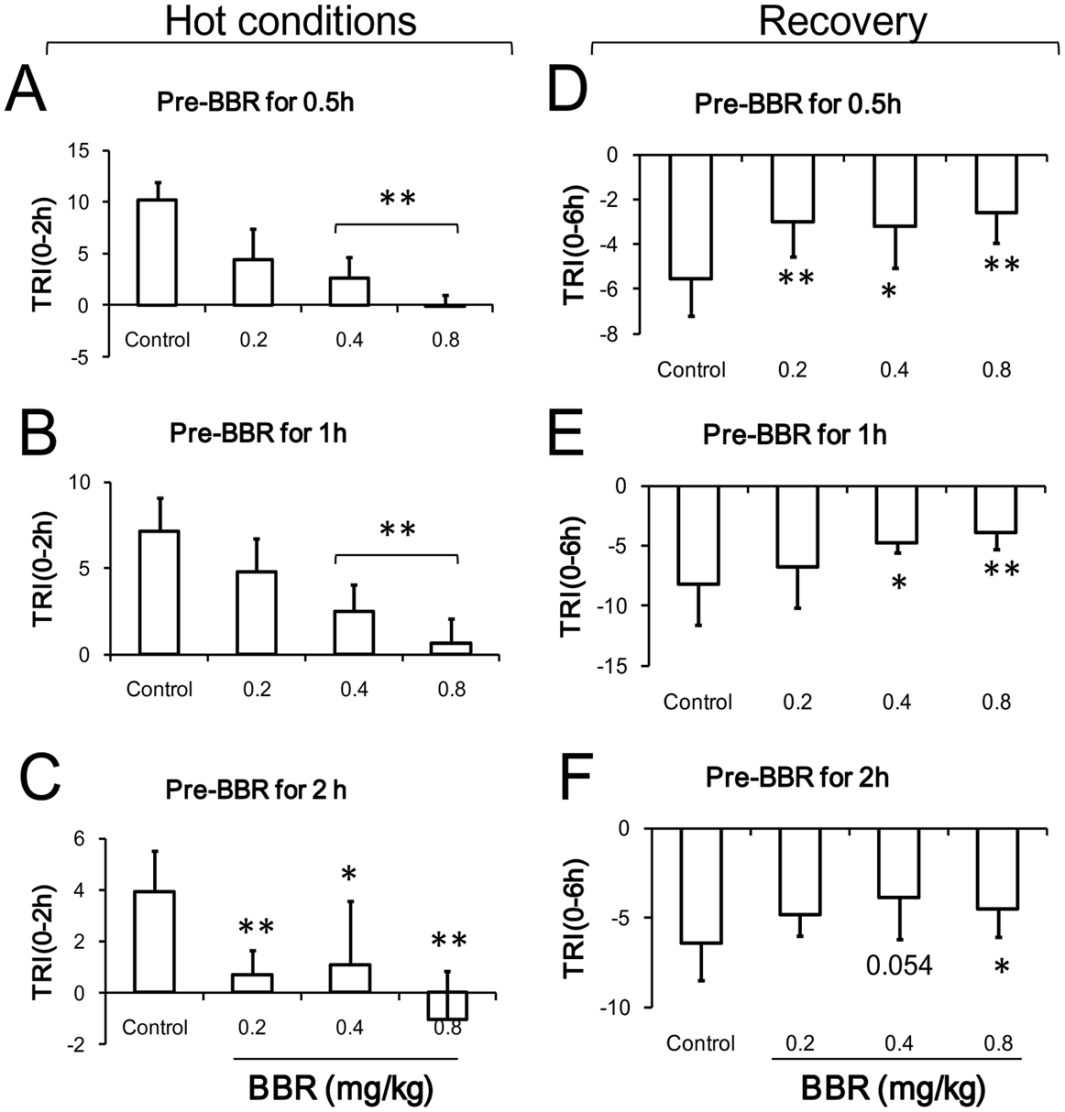
The alteration of core temperature of mice in hot conditions after berberine (BBR) injection. A–C: the data of thermal response index (TRI) during hot conditions (2 hours). A: BBR injection at 0.5 h prior to hot conditioning. F (3, 36) = 40.902, *P*<0.001. B: BBR injection at 1 h prior to hot conditioning. F (3, 36) = 26.683, *P*<0.001. C: BBR injection at 2 h prior to hot conditioning. F (3, 36) = 12.702, *P*<0.001. D–F: the TRI (6 hours) of the recovery after hot conditioning. D: BBR injection at 0.5 h prior to hot conditioning. F (3, 36) = 5.759, *P* = 0.0028. E: BBR injection at 1 h prior to hot conditioning. F (3, 36) = 6.018, *P* = 0.0019. F: BBR injection at 2 h prior to hot stimulation. F (3, 36) = 3.423, *P* = 0.0273. Data were shown as mean ± S.D. from ten independent mice (n = 10). * *v.s.* the control group, *P*<0.05. ** *v.s.* the control group, *P*<0.01.

During the 2 hours of heat conditioning, the TRI of mice in two different dosage groups (0.4 and 0.8 mg/kg) was lower than that of control mice (Figs. 5A–C). Regarding mice treated with BBR 2 hours before exposure to hot conditions, the TRI of mice given 0.2 mg/kg BBR was much lower than that of control mice. After the heat conditioning, the mice were maintained at room temperature for recovery. During the 6 hours of recovery, the TRI of all BBR treated mice remained higher than that of control mice. Among mice treated with BBR 0.5 hours prior to heat conditioning, TRI of mice in all three dosage groups (0.2, 0.4, and 0.8 mg/kg) was significantly higher than that of control mice (Fig. 5D). However, among mice injected 1 and 2 hours before heat conditioning, the TRI of mice given 0.4 and 0.8 mg/kg was higher than that of control mice; the TRI of mice given 0.2 mg/kg was not higher (Figs. 5E–F). This suggests that 6 hours is sufficient time for BBR to affect heat conditioning.

**Figure 6:**
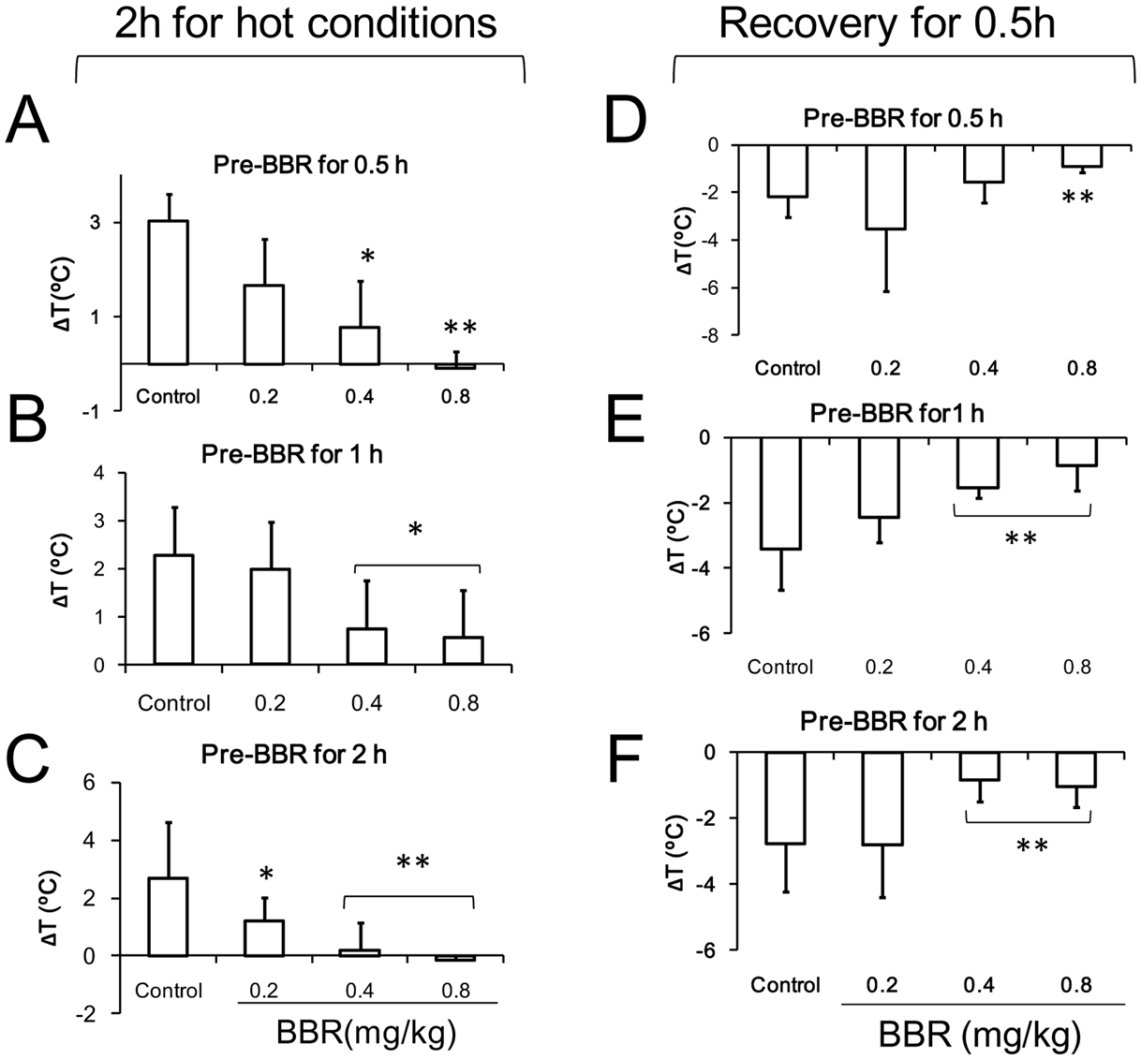
The alteration of core temperature of mice in the course of hot conditions after berberine (BBR) injection. A–C: 2 hour time-point after hot conditioning. A: F (3, 36) = 25.901, *P*<0.001. B: F (3, 36) = 7.922, *P*<0.001. C: F (3, 36) = 15.916, *P*<0.001. D–F: half hour time-point of recovery (post-hot conditioning). D: F (3, 36) = 5.952, *P* = 0.002. E: F (3, 36) = 19.09, *P*<0.001. F: F (3, 36) = 8.545, *P*<0.001. Data were presented as mean ± S.D. from ten independent mice (n = 10).

During the first two hours when the mice situated in the hot environment, BBR dose-dependently decreased the animal’s body temperature (Figs. 6A–C). Compared to injection time-points at 0.5 and 1 h prior to heat exposure, injection of BBR at least 2 hours before exposure to hot conditions augmented the antagonistic effect of BBR on the increased body temperature within all three dosage groups (0.2, 0.4 and 0.8 mg/kg) used in this study. Furthermore, during the recovery process after heat conditioning, the body temperature of mice decreased to a lower level than normal baseline level. BBR at the dosage of 0.4 mg/kg and 0.8 mg/kg can antagonize the decrease in body temperature (Figs. 6D–F), and the time-points of drug administration (0.5, 1, and 2 hours) prior to heat conditioning are independent of this antagonistic effect of BBR.

### Thermoregulation under Cold Conditions

**Figure 7:**
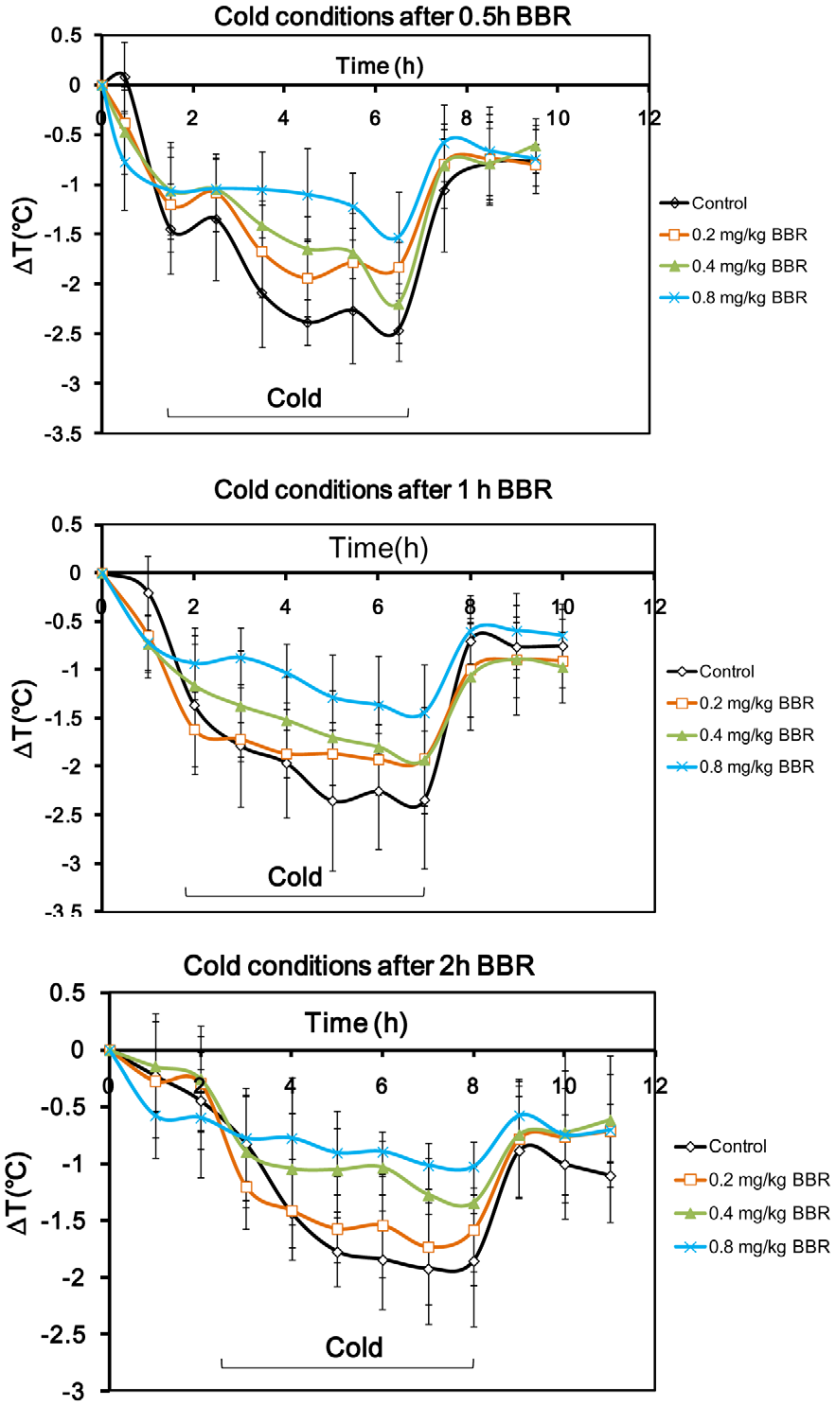
Time course of the temperature of mice after berberine (BBR) injection in cold conditions. Data were presented as mean ± S.D. from ten independent mice (n = 10).

The body temperature of mice under controlled cold conditions (4°C) decreased strikingly. During the 6 hours of cold conditioning, BBR at all three doses (0.2 mg/kg, 0.4 mg/kg, and 0.8 mg/kg) antagonized the body temperature drop. After cold conditioning, the mice were fed under room-temperature (25°C), and the body temperature of mice in all groups recovered quickly. No difference between BBR-administered and control groups was evident (Figs. 7A–C).

**Figure 8:**
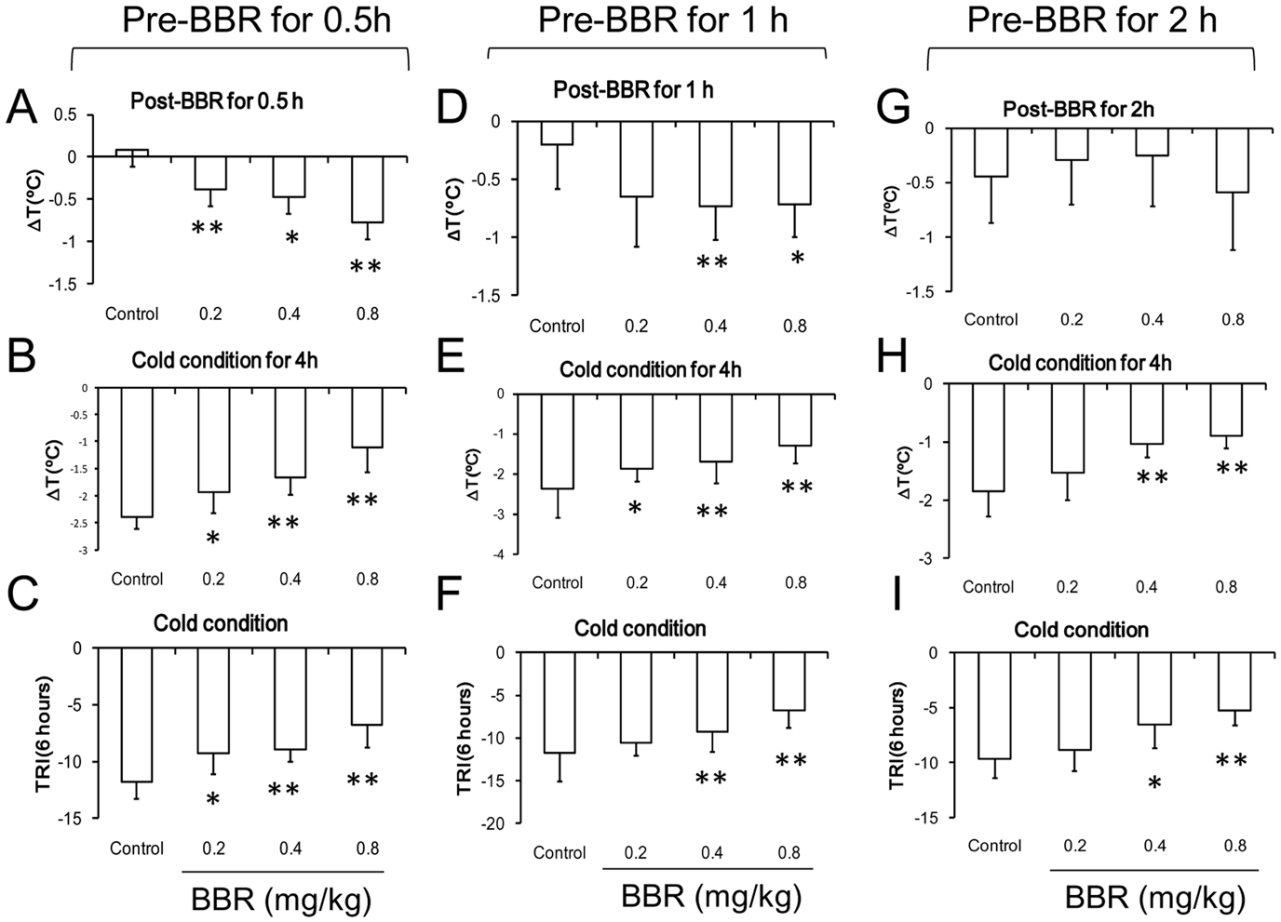
Core temperature of mice in the course of cold conditions after berberine (BBR) injection. A–C: the data for BBR injection at 0.5 h before the experiment. A: temperature of the mice 0.5h after BBR injection at room temperature. F (3, 36) = 7.531, *P* = 0.001. B: temperature of the mice after 4hours in cold conditions. F (3, 36) = 21.82, *P*<0.001. C: The thermo-response index (TRI) in the course of cold conditions. F (3, 36) = 15.821, *P*<0.001. D–F: the data for BBR injection at 1 h before the experiment. D: temperature of normal mice 1 h after BBR injection at room temperature. F (3, 36) = 4.995, *P* = 0.005. E: temperature of the mice after 4 hours in cold conditions. F (3, 36) = 7.089, *P*<0.001. F: the thermo-response index (TRI) in the course of cold conditions. F (3, 36) = 8.048, *P*<0.001. G–I: the data for BBR injection at 2 h before the experiment. G: temperature of normal mice 2 h after BBR injection at room temperature. F (3, 36) = 1.141, *P*>0.05. H: temperature of the mice after 4 hours in cold conditions. F (3, 36) = 15.691, *P*<0.001. I: the thermo-response index (TRI) in the course of cold conditions. F (3, 36) = 12.678, *P*<0.001. Data were shown as mean ± S.D. from ten independent mice (n = 10). * *v.s.* the control group, *P*<0.05. ** *v.s.* the control group, *P*<0.01.

BBR was found to decrease the temperature 0.5 h after injection in a dose-dependent manner (Fig 8 A). One hour after injection, BBR at the dosages of 0.4 and 0.8 mg/kg decreased the normal temperature. Two hours after the injection, no effect of BBR on the body temperature was observed (Figs 8 D and G.). Four hours during cold conditioning, the body temperature decrease was suppressed by BBR in a dose-dependent manner, which was evident irrelevant to the time of BBR administration before cold conditioning (Figs. 8 B, E, and H). During the 6-hour cold conditioning, TRI remained higher than that of control mice at all three different dosages (0.2, 0.4, and 0.8 mg/kg) (Figs. 8 C, F, and J) suggesting that BBR can prevent temperature attenuation caused by cold conditions.

### Thermo-balance between Hot and Cold Conditions

**Figure 9:**
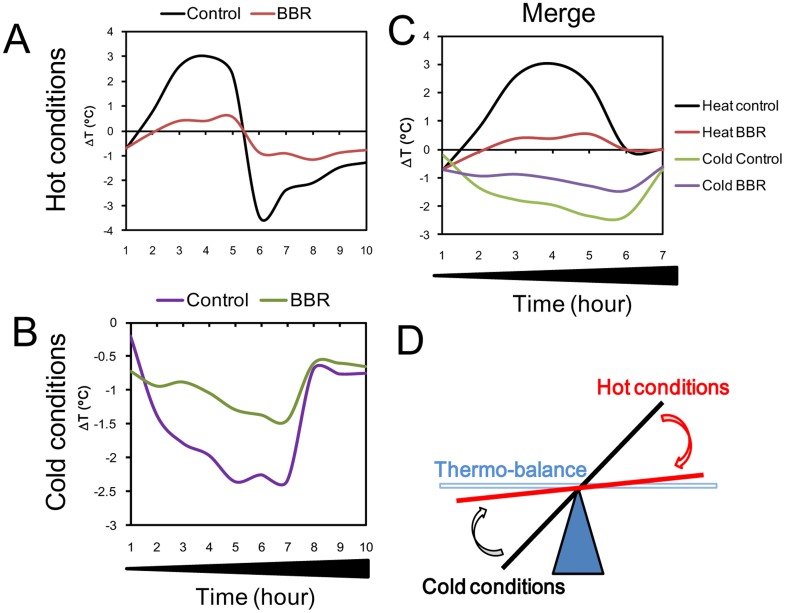
Schematic diagram summarizing the proposed balance of thermal regulation during hot and cold conditions induced by berberine (BBR). A: In hot conditions (40°C). B: In cold conditions (4°C). C: Combination of hot and cold conditions. D: Illustration of thermal balance of BBR in hot and cold conditions.

Under hot conditions (40°C), mouse body temperature increased dramatically and decreased sharply early during the recovery period (25°C). BBR was able to antagonize the changes in body temperature of mice, maintaining it around near normal temperatures.BBR also increased the body temperature to near normal levels during the cold condition, and maintained relatively low body temperature in the hot condition (Figs. 9A–C).

### HSP70, TNFα and TRPM8 Expressions

**Figure 10:**
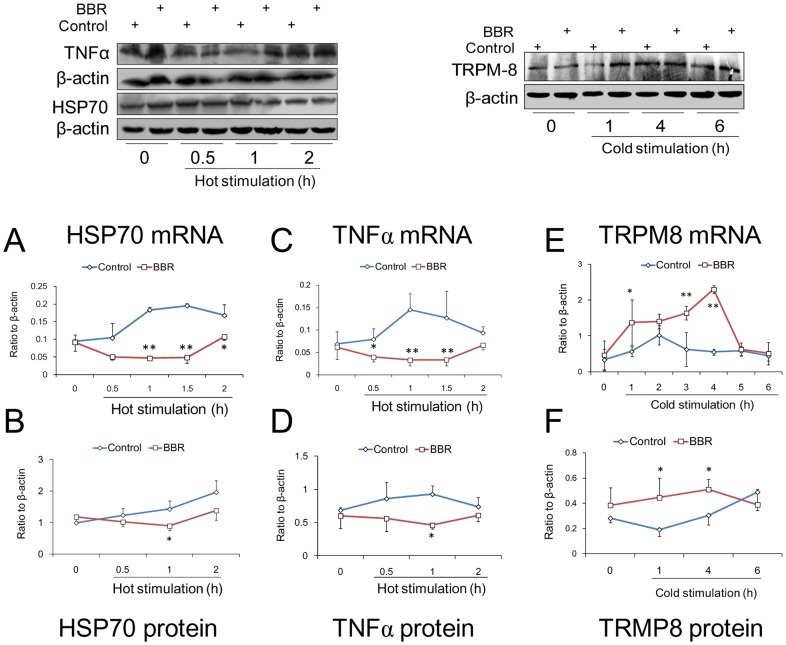
The effect of berberine (BBR) on the expression of HSP70, TNFα and TRPM8 of mouse hypothalamus in hot and cold conditions. The expression of mRNA was detected by real time PCR assay. The expression of protein was determined by Western blot assay. Zero (0) means the time before hot or cold conditioning. 1 hour after intravenous injection of BBR (0.8 mg/kg, i.v.), mice were kept in hot (40°C) or cold (4°C) conditions. A and B depict the expression of mRNA and protein of HSP70 in hot conditions. C and D depict the expression of mRNA and protein of TNFα in hot conditions. E and F depict the expression of mRNA and protein of TRPM8 in cold conditions. Data were shown as mean ± S.D. from 6 independent mice. * *v.s.* the control group, *P*<0.05. ** *v.s.* the control group, *P*<0.01.

The expression of mRNA and protein of HSP70 and TNFα was noticeably enhanced under hot conditions (40°C). The expression of HSP70 did not increase during the first 0.5 hour of heat stimulation, but increased markedly from one hour of heat stimulation and maintained itself at a high level until the end of heat stimulation. The regulation of HSP70 expression during this process in mRNA levels was in good accordance with its protein levels (compared to 0 hour, *P* = 0.034). The regulation of TNFα expression in mRNA level was similar to that of HSP70. After one hour of heat stimulation, TNFα expression was increased and then reduced gradually. However, the protein level of TNFα responded poorly to hot stimulation. There was only a trend towards an increase in TNFα protein compared to before heat conditioning (1 hour time point, *P* = 0.105). BBR inhibited the expressions of mRNA and protein of both HSP70 and TNFα during hot conditions, suggesting that these two factors are involved in the effect of BBR on high body temperatures induced by hot environments (Figs. 10A–D).

During the first two hours of cold conditions, the mRNA expression of TRPM8 in the control group increased evidently and then decreased to normal levels. However, the protein level of TRPM8 decreased in the first one hour after cold conditioning (compared to 0 hour, *P* = 0.035) and recovered, compared to the onset of cold conditioning (*P* = 0.175), until the end of cold stimulation (6 hours during cold conditioning). This indicates more complicated mechanisms in TRPM8 and temperature response. Therefore, after cold conditioning, only an increase in TRPM8 mRNA levels by BBR was evident, suggesting that TRPM8 is involved in the effect of BBR on cold environmental stimulation (Figs. 10E–F), but a more complicated mechanism exists.

### HSP70 Inhibitor in Hot Conditions

**Figure 11:**
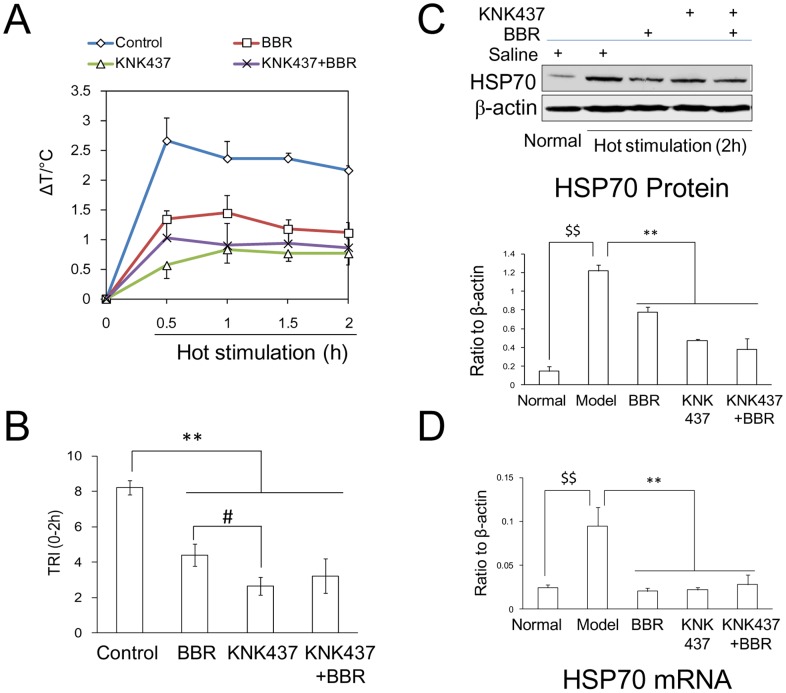
Alteration of mouse core temperature and HSP70 expression by the inhibitor of HSP70. The expression of mRNA was detected by real time PCR assay. The expression of protein was determined by Western blot assay. KNK437, an inhibitor of HSP70, was administered as 50 mg/kg by intraperitoneal injection (i.p.). 1 hour after intravenous injection of berberine (BBR) (0.8 mg/kg, i.v.), mice were kept in hot (40 °C) conditions for 2 hours. A: Time course of *T*r by hot conditioning. B: Temperature response index (TRI) for 2 hours in hot conditions. F (3, 8) = 43.29, *P*<0.001. C: The expression of HSP70 protein. F: (4, 10) = 13.507, *P*<0.001. D: The expression of HSP70 mRNA. F: (4, 10) = 11.701, *P*<0.001. Data were shown as mean ± S.D. from 3 independent mice. * *v.s.* the control group (model), *P*<0.05; ** *v.s.* the control group (model), *P*<0.01. # *v.s.* BBR group, *P*<0.05. $$ *v.s.* normal group, *P*<0.01.

KNK437 is a small molecule inhibitor of HSP70 [28]. After intraperitoneal injection of KNK437 in mice, *T*r did not increased appreciably after heat conditioning. However, the body temperature of mice without KNK437 administration increased by 2°C. Therefore, BBR can attenuate body temperature under hot conditions, but is weaker than KNK437. In KNK437-BBR groups, *T*r decreased evidently to temperatures close to that of the KNK437 groups (Figs. 11 A and B). Accordingly, the distinct expression of both mRNA and protein levels of HSP70 was upregulated in control groups with normal saline after hot conditions. Both KNK437 and BBR can inhibit the increased expression of mRNA and protein levels of HSP70. However, no synergistic effect was found between KNK437 and BBR (Figs. 11 C and D).

### Motor and Exploiting Behavior

**Figure 12:**
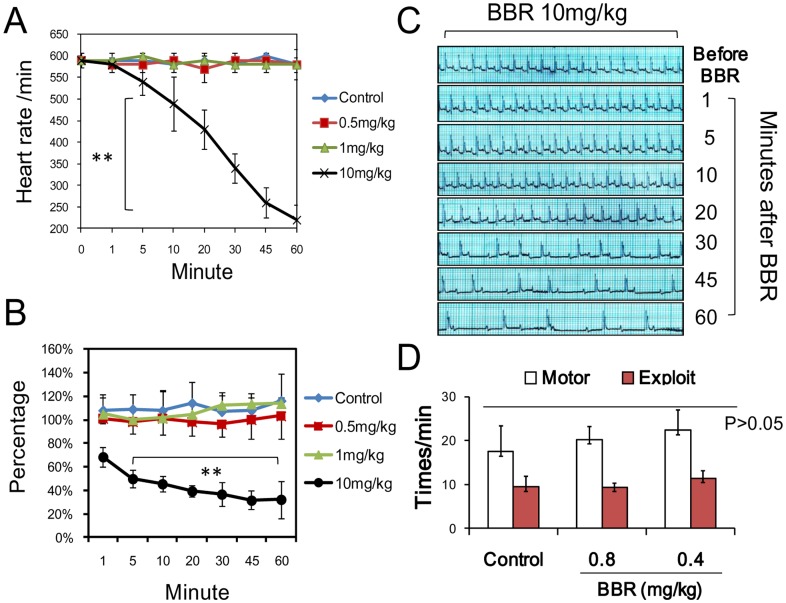
Electrocardiography (ECG) and the motor behavior of mice after berberine (BBR) injection. A depicts the heart rate (beats/min) for three different dosages of BBR (0.5, 1 and 10 mg/kg) (i.v.). F (3, 24) = 9.168, *P*<0.001 (n = 6). B depicts the percentage of cerebral blood flow rate increasing with three different dosages of BBR (0.5, 1 and 10 mg/kg) (i.v.). F (3, 24) = 126.56, *P*<0.001 (n = 6). C is the electrocardiogram of the mouse with BBR dosage of 10 mg/kg. D illustrates the motor activity of mice at two different dosages of BBR (0.8 and 0.4 mg/kg) (i.v.). F (2, 21) = 3.193, *P* = 0.136 (n = 8). Data were shown as mean ± S.D. from 6 to 8 independent mice. ** *v.s.* control groups, *P*<0.01.

BBR was found to reduce heart rate (HR) at a dose of 10 mg/kg (Fig. 12C). However, with the BBR dosages we used (0.5 and 1 mg/kg), no inhibition of mouse heart rate or changes in the ECG relative to the control were observed (Fig. 12A). BBR also could not reduce mouse cerebral blood flow rate at the dosage of 0.5 and 1 mg/kg (Fig. 12B). At the dosage of 10 mg/kg, mouse HR reduced sharply within 5 min after intravenous BBR injection. Then, 60 minutes after drug administration, the mouse HR decreased from 580 beats/minute to 220 beats/minute. The cerebral blood flow rate decreased to 50% compared to the flow rate before BBR administration, which correlates with the reduction in HR. At the dosage of 1 mg/kg, BBR could not change the HR of the mice, indicating that the dosages used in hot/cold conditions were safe and did not affect mouse circulatory function.

The mouse movement and the exploit behavior reflect the function of central nervous system (CNS). No effect of BBR with the dosage of 0.4 and 0.8 mg/kg was observed in this experiment (Fig. 12 D).

## Discussion

Berberine (BBR) is an antibacterial agent and is clinically used to treat intestinal inflammation. The antifebrile effect of BBR is thought to be in part due to some of its antibacterial effects. In our previous studies with BBR, we noticed the effect of BBR on body temperature and hypothesized that BBR could act directly on thermal regulation in vivo. Data from mice under room temperature verified that BBR was able to decrease the body temperature of mice in a dose dependent manner (0.2, 0.4, and 0.8 mg/kg). Physical models of high body temperature and low body temperature in mice induced by controlled environmental temperature were used in the present study to explore the potential effect of BBR on thermal regulation of mice under abnormal temperatures.

In the experimental system, BBR could dose-dependently antagonize the alteration of body temperature under hot or cold conditions and maintain the body temperature close to its baseline, indicating that BBR can balance the mouse body temperature under both hot and cold conditions. After completion of heat conditioning, mouse body temperature decreased immediately and then slowly returned to normal, which is consistent with previous reports [40]–[42], suggesting a need for the physiology of thermoregulatory control to be studied in more detail [43]. BBR was found to prevent the sharp decrease in body temperature in the post-heat conditioning process and promote recovery, maintaining the balance between heat conditioning and recovery. Therefore, thermo-balance via BBR under hot and cold conditions can be concluded (Fig. 9D).

It was reported that the distinct upregulation of HSP70 and TNFα correlate with the response of thermoregulation to heat stimulation [44], [45]. HSP70 was shown to be an intermediary in the thermal effects on TNFα expression, because the use of HSP70 inhibitors significantly eliminated the thermal-dependent enhancement of TNFα expression [22]. The present study found an upregulation of HSP70 and TNFα in the hypothalami of mice exposed to hot conditions; BBR was able to inhibit HSP70 and TNFα expression during heat conditioning, suggesting that the thermal mechanism of BBR correlates with these two factors. During hot conditions, KNK437, an inhibitor of HSP70, was found to clearly inhibit the body temperature, which confirms the previous study that HSP70 is a crucial factor in temperature response by heat stimulation [29]. Simultaneously, BBR was also found to inhibit the response of body temperature to environmental temperature. There was no synergistic effect when BBR was administered with KNK437 because *T*r of the mice was not reduced significantly compared to that of KNK437 groups. The expression of mRNA and protein of HSP70 also decreased in KNK437 groups and BBR groups. We can, therefore, conclude that HSP70 is one of the crucial targets of BBR during hot conditions.

In recent studies, TRPM8 is recognized as a sensor in peripheral nerves for transporting the cold signal to the central nervous system (CNS) [46], [47]. However, there are few reports concerning TRPM8 and the hypothalami of mice during cold conditions. In our study, TRPM8 in control mice was found to be distinctly upregulated in mRNA levels after 2 hours in cold conditions and then decreased close to normal; however, the protein levels of TRPM8 were not responsive to cold stimulation as mRNA was, suggesting that there was a suppression of upregulation of TRPM8 protein during cold conditions. BBR was found to upregulate TRMP8 expression in both mRNA and protein levels, suggesting that the mechanism of thermal regulation via BBR under cold conditions is pertinent in affecting TRPM8 levels. Therefore, TRPM8 was considered as a potential target of BBR in mouse body temperature attenuation under cold conditions.

Normally, environmental hot and cold conditions was found to cause cutaneous thermal receptor to respond and send feed-forward afferent signals to brain thermo-sensitive neurons in hypothalamus [37]. They, in turn, activate specific populations of sympathetic and somatic afferents [46]. Under cold conditions, heat production in the body requires thermogenesis. While under hot conditions, heat loss fosters thermal balance. BBR disrupted both hot and cold responses, implying that it must target crucial factors by preventing the feed-forward afferent signals from activating the thermo-sensitive neurons. HSP70 is recognized as a factor in stress response to cell survival protection as well as in cell thermal response [48]. TRPM8 is recognized as the cold sensor for afferent signals to thermo-sensitive neurons. The results of HSP70, TNFα and TRPM8 in the hypothalami demonstrate that BBR may exert its thermal regulative effects by acting on these factors, interfering with the response of body temperature to the environmental change.

Regarding the toxicity of BBR, we conducted the ECG and the motor behavior experiments with mice. In ECG, 1 mg/kg of BBR did not influence mouse heart ECGs, indicating that this dose is safe for heart function. Our previous work suggests that the LD_50_ of BBR was at 9.0386 mg/kg by intravenous injection [30]. A dose of 10 mg/kg of BBR was found to inhibit the heart rate, which supported the previous LD_50_ conclusion. It has been reported that the depression of the CNS, such as that caused by diazepam, can reduce the stress-induced hyperthermic (SIH) response in rodents [49], and the motor movement and the exploit behavior are often used as an index of mouse CNS function [50]. The ECG and motor experiments confirmed that there is no such interference at any BBR dosage in our experiments.

In conclusion, BBR can influence the body temperature of mice and affect thermal regulation under hot and cold conditions. HSP70, TNFα and TRPM8 in the hypothalamus are involved in the mechanism of BBR thermal regulation. Since HSP70, TNFα and TRPM8 have not been thoroughly understood regarding their effects on thermoregulation, there are many challenges for comprehending the fundamental specificities of BBR that affect expression of these factors. Further research is, therefore, warranted to address these issues in more detail.
